# Diagnostic Challenge for Positive 1,3-β-D-Glucan in an Immunocompromised Patient Receiving Intravenous Immunoglobulin Presenting With Respiratory Failure

**DOI:** 10.7759/cureus.61121

**Published:** 2024-05-26

**Authors:** Seohyeon Im, Estefany Garces, Timothy Roedder, William Charini

**Affiliations:** 1 Internal Medicine, Mass General Brigham-Salem Hospital, Salem, USA; 2 Pulmonary and Critical Care, Mass General Brigham-Salem Hospital, Salem, USA; 3 Infectious Disease, Mass General Brigham-Salem Hospital, Salem, USA

**Keywords:** immunocompromised hosts, immunocompromised, false-positive diagnosis, fungal lung infection, pneumocytis jiroveci, beta-d glucan

## Abstract

Diagnosing *Pneumocystis jirovecii* pneumonia (PJP) can be complex, particularly in cases of significant respiratory failure. The 1,3-β-D-glucan (BDG) serum assay has emerged as a promising non-invasive diagnostic tool for detecting fungal infections, including PJP. However, factors that can confound the interpretation of BDG levels by causing elevation in serum levels have been documented. Here, we present the case of 51-year-old woman with underlying autoimmune disorder, hematologic malignancy, and chronic steroid use, who was admitted for acute hypoxemic respiratory failure. Obtaining the BDG assay after the administration of intravenous immunoglobulin (IVIG) posed a diagnostic challenge, as the patient was unable to undergo bronchoscopy. This circumstance led to a debate regarding the possibility of a false-positive BDG due to IVIG use or the presence of PJP. Ultimately, the patient was empirically treated for PJP. This case underscores the importance of comprehending factors that may contaminate BDG results, particularly in immunocompromised individuals.

## Introduction

This case was previously presented as a poster at American College of Physicians Massachusetts Chapter 2023 Annual Scientific Meeting on September 23, 2023, in Waltham, Massachusetts.

1,3-β-D-glucan (BDG) is a cell wall component of numerous fungal organisms, and its assay can detect fungal infections such as invasive aspergillosis, invasive candidiasis, and *Pneumocystis jirovecii* pneumonia (PJP) [1]. This nonmolecular fungal marker can be utilized to support the diagnosis of PJP when invasive procedures are restricted or contraindicated [1]. However, false-positive results have been reported with certain antibiotics, bacteremia, cellulose-related iatrogenic contamination, and blood-derived products including intravenous immunoglobulin (IVIG), leading to diagnostic challenges, particularly in immunocompromised individuals [1-3].

## Case presentation

A 51-year-old woman presented with worsening dyspnea on exertion over the past few days. She also experienced general weakness with difficulty navigating stairs for the past week. She denied fever, cough, sputum production, or chest pain and breathed comfortably at rest.

The patient had a history of systemic sclerosis complicated by interstitial lung disease (ILD), which had recently flared, leading to hospitalization five weeks before admission. Upon discharge, she was prescribed a prednisone taper starting at 60 mg daily and mycophenolate mofetil, which had been resumed after a three-month hold. She was also started on trimethoprim/sulfamethoxazole (TMP/SMX) 400/80 mg daily for PJP prophylaxis due to high-dose steroids. Additionally, she had recently been diagnosed with multiple myeloma and had completed her fourth cycle of induction therapy (daratumumab and bortezomib) 15 days prior to admission. At the time of admission, she was on prednisone 20 mg daily.

Upon admission, the patient presented with an oxygen saturation of 85% on room air, which promptly improved with 2 L/min of oxygen via nasal cannula. She was afebrile. Physical examination revealed intermittent end-expiratory rhonchi on lung auscultation and proximal muscle weakness with 4/5 strength.

**Figure 1:**
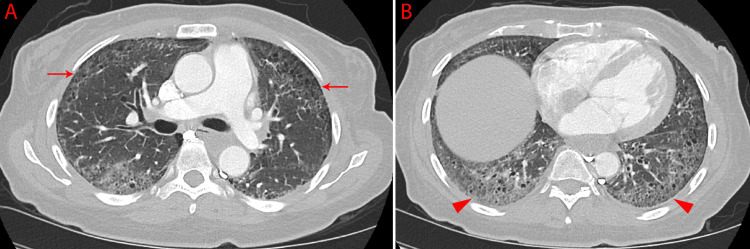
A CT scan of the chest, axial view—A) bilateral peripheral traction bronchiectasis (arrows); B) bilateral peripheral traction bronchiectasis with extensive ground-glass opacification (arrow heads)

Laboratory investigations were notable for high level of creatinine kinase (CK) at 3,203 U/L (reference range 29-201 U/L), but indicated normal levels of white blood cells, erythrocyte sedimentation rate, and C-reactive protein. CT chest pulmonary angiogram excluded pulmonary embolism and demonstrated bilateral peripheral traction bronchiectasis with ground-glass opacities with a basilar predominance (Figure 1). This was consistent primarily with ILD and resembling findings from prior CT scans.

The acute hypoxemic respiratory failure was attributed primarily to the exacerbation or progression of her underlying ILD. Although PJP remained a possibility, it was deemed less likely due to her PJP prophylaxis.

IV methylprednisolone was initiated, and a BDG assay was ordered to rule out PJP. Concurrently, IVIG was administered to address muscle weakness and elevated CK, suggestive of inflammatory myopathy. Eventually, the BDG sample, obtained one hour post-IVIG administration from the same arm, returned >500 pg/ml (Fungitell® assay, reference range < 60 pg/ml). While it was recognized that IVIG could potentially cause false-positive BDG results, the infectious process could not be entirely excluded.

The patient's significant desaturation with movement posed a high risk for bronchoscopy, so bronchoalveolar lavage (BAL) for a definitive diagnosis was not performed. Instead, full treatment dose of TMP/SMX (15 mg/kg/day TMP component) was empirically started. The patient was discharged with a total 21-day course of full-dose TMP/SMX and prednisone taper.

The Pneumocystis polymerase chain reaction (PCR), obtained at the onset of full-dose TMP/SMX therapy, returned negative 10 days after, but she completed the rest of the course as prescribed to treat in cautious way. Subsequently, a repeat of the BDG assay was attempted following the antibiotic course, but unfortunately, it yielded an uninterpretable result due to hemolysis.

## Discussion

*Pneumocystis jirovecii*, an opportunistic pathogen, is the causative agent of PJP. Historically associated with HIV/AIDS, PJP has traditionally been diagnosed in HIV-positive individuals with CD4+ T lymphocyte counts < 200 cells/mm3. However, its incidence has broadened to include non-HIV patients with compromised cell-mediated immunity due to factors such as hematologic malignancies, chemotherapy, solid organ or hematopoietic stem cell transplantation, or immunosuppressive therapy for autoimmune disorders [4]. 

*Pneumocystis jirovecii* has proven to be extremely difficult to culture in vitro [5]. Thus, diagnosing PJP conventionally relies on detecting the pathogen in respiratory samples from patients. BAL typically offers the greatest diagnostic yield, although induced sputum and oropharyngeal wash samples are also utilized. While the traditional Gomori-methenamine silver (GMS) stain has been the standard method, studies have indicated that immunofluorescent staining exhibits higher sensitivity and specificity [5]. PCR of respiratory samples has also demonstrated higher sensitivity compared to conventional staining techniques, although false positive results were reported, likely attributable to colonization [6].

Recently, the BDG assay has emerged as a valuable alternative for diagnosing fungal infections, including PJP, particularly in severely ill patients who are unable to undergo bronchoscopy [1]. BDG, a polysaccharide present in the cell wall of most pathogenic fungi (excluding Mucorales), serves as the target for this assay [1]. Notably, the serum assay is not suitable for detecting Cryptococcus spp. and Blastomyces dermatitis due to their minimal release of BDG [2].

A meta-analysis revealed the BDG assay to possess a sensitivity of 94.8% and specificity of 86.3% for diagnosing PJP, demonstrating its efficacy in both HIV-infected and non-HIV immunocompromised populations [7]. In a retrospective study involving patients with underlying malignancies, a serum BDG level < 80 pg/ml exhibited a high negative predictive value of 95.2%. Furthermore, in patients with positive Pneumocystis PCR in BAL, a high serum BDG level > 200 pg/ml strongly suggested infection rather than colonization [8].

However, several documented factors can lead to false-positive results in BDG testing, complicating interpretation (Table 1). These factors include the administration of blood-derived products, therapeutic antibodies, certain bacterial infections, and antibiotics [1-3]. One reason for false positivity is the presence of cellulose, a component of plant cell walls that primarily contains β (1,4) D-glucan linkages [7,9]. The use of cellulose membranes in filters for IVIG manufacturing is thought to contribute to false positivity in patients receiving IVIG [2]. Other blood products, such as packed red blood cells and human albumin, have also been associated with false-positive BDG results [10]. This was previously investigated by in vitro experiments demonstrating BDG release from the depth filters used in blood clarification processes [11]. Additionally, gauze packing of serosal surfaces during surgery has also been noted to elevate serum BDG levels [7]. Renal replacement therapy was previously connected to elevated BDG levels [12]. But modern non-BDG-leaching membranes have largely replaced cellulose dialysis membranes, mitigating this issue [3]. False-positive BDG assay results related to bacteremia from gram-negative bacilli, *Pseudomonas aeruginosa*, and nocardiosis have been reported in studies with small populations [13-15]. However, their significance remains controversial due to variability in findings [2,16] and the lack of well-demonstrated BDG presence from bacteria [3]. The contamination with various antibiotics has been observed in in vitro studies [17]. However, its likelihood is considered lower due to the high dilution ratio during IV injection [2,3].

**Table 1:** Factors affecting BDG elevation IG: Immunoglobulin; BDG: β-D-glucan

Factor	Notes
IG	Strong association with BDG elevation [2,18,19], likely due to contamination during production [2].
Other blood-derived products	Includes packed red blood cells and human albumin [10], likely due to depth filter use [11].
Therapeutic antibodies	The pool of monoclonal/polyclonal antibodies showed significant association with BDG elevation in a study [2].
Gauze packing	Gauze packing of serosal surfaces during surgical procedures may result in the release of BDG [7].
Renal replacement therapy	Historically related to BDG elevation [12]. The impact has decreased with the advancement of dialysis membranes [3].
Bacterial infections	Gram-nagative bacilli , Pseudomonas aegurinosa, and nocardiosis have been reported in the literature, but their significance is controversial [2,13-16].
Antibiotics	Multiple antibiotics have been observed to elevate BDG levels in vitro [17].

The contributory role of IVIG to false-positive BDG results is well established in the literature. In a study involving patients with hematologic malignancy, IVIG administration was strongly linked to false-positive, with an odd ratio of 7.8 [2]. Moreover, very high levels of BDG above 523 pg/ml were detected in 100 % of patients (28/28) who received IVIG [18]. A study in pediatric hemato-oncologic patients showed peak BDG levels within 3 days after IVIG infusion, with BDG levels normalizing in 64.0%, 76.5%, and 100% of patients on days 7, 14, and 21 after IVIG infusion, respectively [19].

Applying these findings to our case, we hypothesize that IVIG infusion and laboratory drawing from the same side of the arm contributed to a false-positive BDG result.

## Conclusions

This case emphasizes the complexity of whether to treat PJP empirically or not in the context of immunocompromised patients with respiratory failure and IVIG use. Patients who receive IVIG are highly likely to be in a condition of immunodeficiency and vulnerable to opportunistic infections. At the same time, IVIG can cause false positive serum BDG results. Thus, clinicians must be cautious interpreting BDG assay results in this setting.
